# Supplementary material for: Measurement and classification of bold–shy behaviours in medaka fish
Source: Bioinformatics. 2025 Jun 12;41(6):btaf342. doi: 10.1093/bioinformatics/btaf342 (PMC12206527; doi:10.1093/bioinformatics/btaf342)
Supplement: btaf342_Supplementary_Data [file btaf342_supplementary_data.pdf]

Measurement and classification of bold-shy behaviours in medaka fish

Saul Pierotti*[1], Ian Brettell*[1], Tomas Fitzgerald[1], Cathrin Herder[2], Narendar Aadepu[2], Christian Pylatiuk[3], Joachim Wittbrodt[4], Ewan Birney[1], Felix Loosli†[2]

[1] European Molecular Biology Laboratory, European Bioinformatics Institute, Wellcome Genome Campus, Hinxton, Cambridge, UK

[2] Institute of Biological and Chemical Systems, Biological Information Processing (IBCS-BIP), Karlsruhe Institute of Technology, 76131 Karlsruhe, Germany

[3] Institute for Automation and Applied Informatics (IAI), Karlsruhe Institute of Technology, 76131 Karlsruhe, Germany

[4] Centre for Organismal Studies, Heidelberg University, Im Neuenheimer Feld, Heidelberg, Germany

* These authors contributed equally to this work.

† To whom correspondence should be addressed. Email: felix.loosli@kit.edu

Supplementary Notes

Positioning of the HMM states along the bold-shy behavioural axis

In the main test we describe the general principles that we followed in positioning HMM states along the bold-shy behavioural axis. Using these criteria, we conclude that HMM states 1 and 4 indicate shy behaviour as they are more frequent at the beginning of the videos (both in the open field as well as the novel object tests) and are associated with low levels of locomotion (**Figure S1** and **S2**). State 9 indicates a bold state as its frequency increases over time and is associated with high levels of locomotion encompassing the entire tank area. Similarly states 5, 6 and 10-12 show high locomotion activity in all tank areas, though with a less pronounced temporal increase of frequency. States 13-15 are more ambiguous in that they are associated with high locomotion and a slight temporal increase. However, tank positions are mostly at the perimeter. Furthermore, the frequency reaches an early peak at about 100 seconds followed by a gradual decrease. This is reminiscent of the behavioural pattern observed with *Kaga* in the open field test where initially high locomotion activity subsequently decreases (**Figure 5**). We therefore conclude that this state can indicate stress related high activity. The occupancy of state 7 and 8 increases over time and is associated with moderate locomotion and high occupancy of corner regions, suggesting exploratory behaviour of these regions. Finally states 2 and 3 exhibit slow locomotion and corner occupancy which could indicate a cautious resting state.

Supplementary Tables

Table S1: Strain-dependent differences and variance explained from the ANOVA test for the proportion of time spent in each HMM state by the test fish (direct genetic effect) across test fish strains for the open field and novel object assay components.

Assay component	HMM State	*p*-value (FDR-adjusted)	Significance	Variance explained
Open field	1	1.17e-24	****	0.297
Open field	2	4.75e-07	****	0.101
Open field	3	6.42e-07	****	0.0977
Open field	4	6.06e-21	****	0.261
Open field	5	2.36e-02	*	0.0358
Open field	6	1.73e-02	*	0.0384
Open field	7	5.54e-07	****	0.0964
Open field	8	3.76e-05	****	0.0699
Open field	9	8.54e-05	****	0.0673
Open field	10	3.80e-01	ns	0.0161
Open field	11	2.36e-01	ns	0.0209
Open field	12	3.11e-01	ns	0.0185
Open field	13	5.35e-03	**	0.0452
Open field	14	2.01e-02	*	0.0406
Open field	15	1.98e-01	ns	0.0221
Novel object	1	2.79e-13	****	0.175
Novel object	2	1.53e-07	****	0.109
Novel object	3	2.25e-07	****	0.106
Novel object	4	5.85e-10	****	0.139
Novel object	5	1.48e-04	***	0.0681

Novel object	6	1.56e-04	***	0.0681
Novel object	7	2.25e-07	****	0.103
Novel object	8	7.52e-01	ns	0.00636
Novel object	9	2.01e-06	****	0.0944
Novel object	10	2.93e-01	ns	0.019
Novel object	11	3.87e-01	ns	0.0154
Novel object	12	4.75e-01	ns	0.0135
Novel object	13	3.62e-01	ns	0.0148
Novel object	14	8.68e-01	ns	0.00513
Novel object	15	6.97e-01	ns	0.00938

Table S2: Strain-dependent differences and variance explained from the ANOVA test for the proportion of time spent in each HMM state by the reference fish (social genetic effect) across test fish strains for the open field and novel object assay components.

Assay component	HMM State	p-value (FDR-adjusted)	Significance	Variance explained
Open field	1	2.99e-05	****	0.0815
Open field	2	8.81e-01	ns	0.00361
Open field	3	8.91e-01	ns	0.0031
Open field	4	1.58e-05	****	0.0864
Open field	5	4.74e-01	ns	0.0102
Open field	6	4.45e-01	ns	0.0108
Open field	7	6.82e-01	ns	0.00696
Open field	8	2.40e-01	ns	0.0169
Open field	9	6.74e-01	ns	0.00709
Open field	10	2.02e-01	ns	0.021
Open field	11	1.14e-01	ns	0.0269
Open field	12	9.43e-02	ns	0.0285
Open field	13	1.29e-01	ns	0.0233
Open field	14	1.60e-01	ns	0.0244
Open field	15	6.85e-01	ns	0.00719
Novel object	1	3.07e-05	****	0.0774
Novel object	2	1.15e-01	ns	0.0265
Novel object	3	1.15e-01	ns	0.0263
Novel object	4	1.90e-05	****	0.0815
Novel object	5	2.88e-01	ns	0.0165
Novel object	6	2.53e-01	ns	0.018
Novel object	7	1.54e-02	*	0.0393
Novel object	8	5.62e-02	ns	0.0292
Novel object	9	2.35e-01	ns	0.0203
Novel object	10	2.40e-01	ns	0.0199
Novel object	11	1.60e-01	ns	0.0246
Novel object	12	1.62e-01	ns	0.0243
Novel object	13	2.31e-01	ns	0.0182
Novel object	14	2.38e-01	ns	0.0191
Novel object	15	4.26e-01	ns	0.0138

Table S3: Strain-dependent differences in the co-occupancy of the same HMM state by the test and reference fish, stratified by assay component and HMM state.

Assay component	HMM State	p-value (FDR-adjusted)	Significance
Open field	1	1.68e-07	****
Open field	4	7.98e-06	****
Open field	7	1.30e-02	*
Open field	2	1.30e-02	*
Open field	3	8.03e-03	**
Open field	6	5.42e-01	ns
Open field	5	6.85e-01	ns
Open field	8	2.82e-01	ns
Open field	9	3.69e-01	ns
Open field	12	3.07e-01	ns
Open field	11	2.82e-01	ns
Open field	10	4.83e-02	*
Open field	13	7.30e-01	ns
Open field	14	7.83e-01	ns
Open field	15	1.30e-02	*
Novel object	1	2.54e-07	****
Novel object	4	4.79e-07	****
Novel object	7	8.03e-03	**
Novel object	2	1.30e-02	*
Novel object	3	4.83e-02	*
Novel object	6	9.10e-02	ns
Novel object	5	9.10e-02	ns
Novel object	8	7.76e-01	ns
Novel object	9	9.10e-02	ns
Novel object	12	7.54e-01	ns
Novel object	11	7.54e-01	ns
Novel object	10	8.80e-01	ns
Novel object	13	7.76e-01	ns
Novel object	14	5.66e-01	ns
Novel object	15	3.64e-01	ns

Supplementary Figures

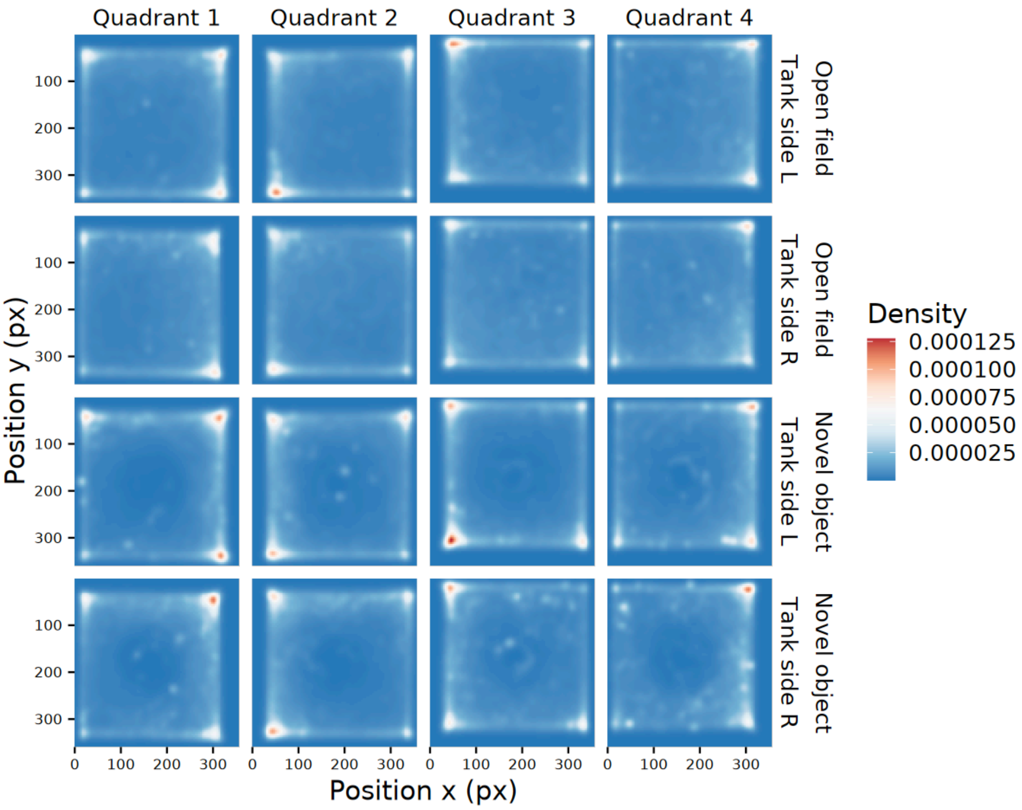

Figure S1: Areas of the test tanks occupied by the fishes stratified by assay component, quadrant, and tank side (left or right testing apparatus).

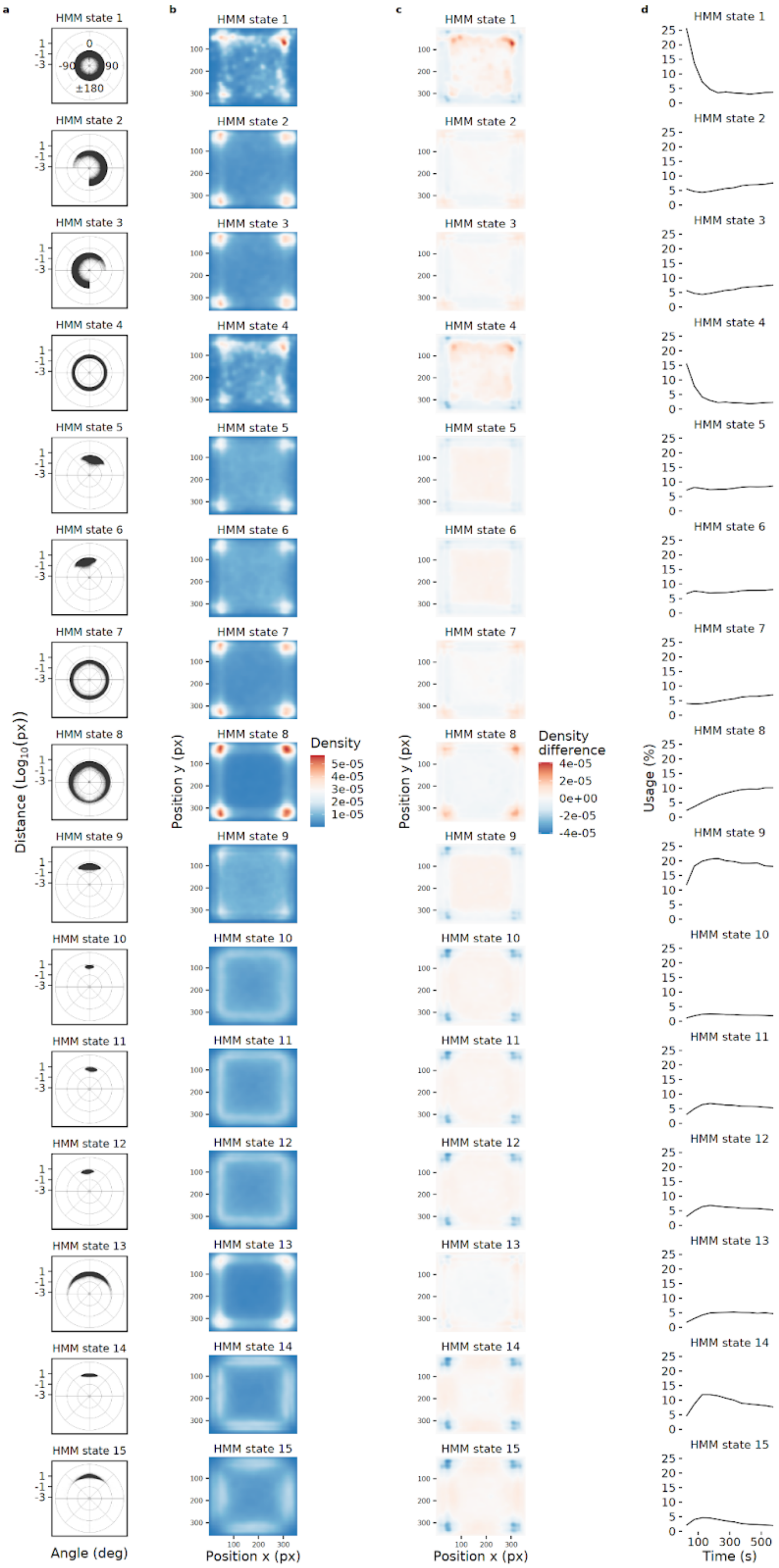

Figure S2: Characterization of the hidden states in the open field assay component with respect to distances and angles (a), position of the fish in the tank (b), difference in the density of fish positions in the tank with respect to the fish position density calculated excluding the first 400 seconds of habituation (c), and hidden state usage across time (d).

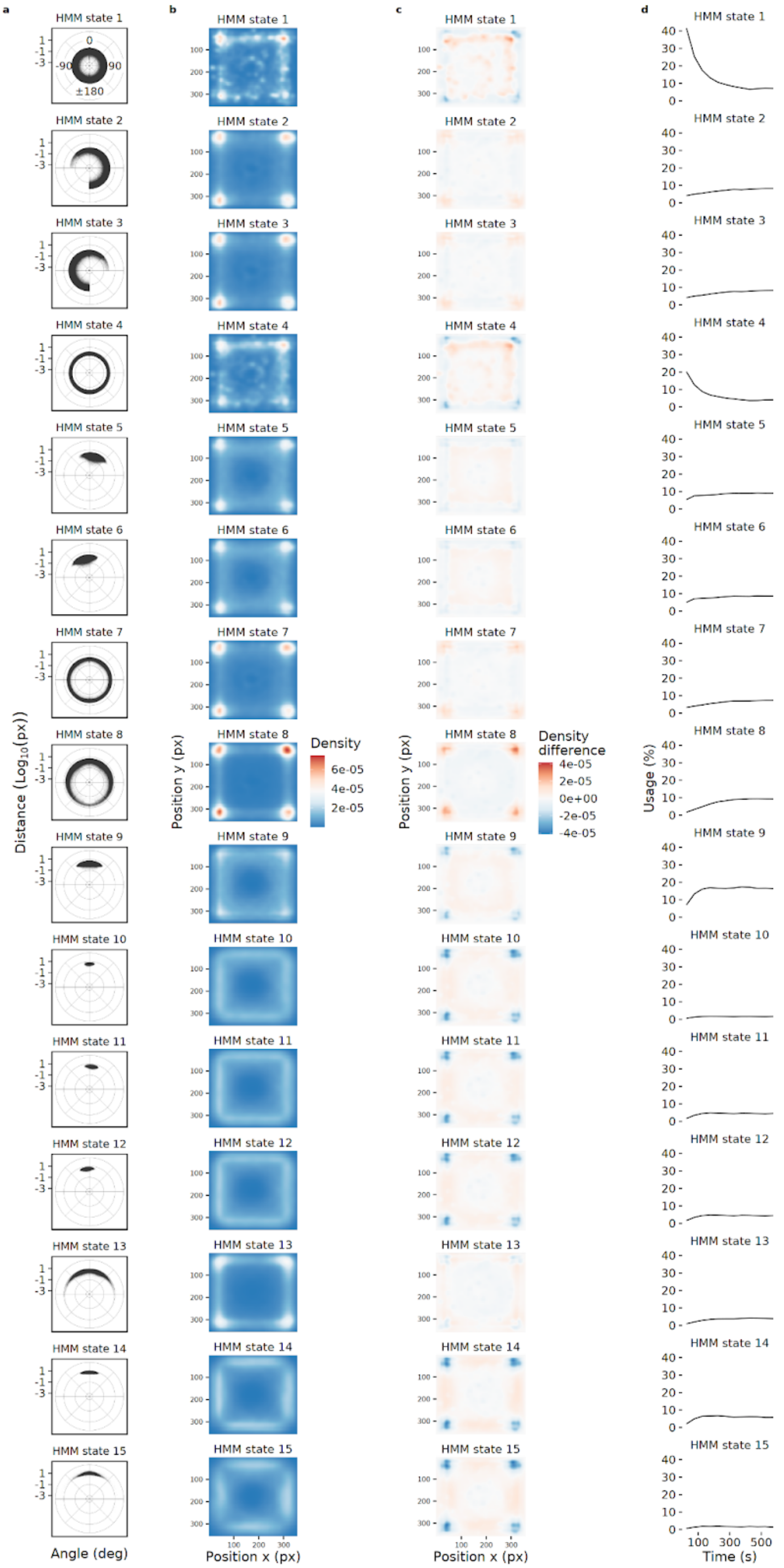

Figure S3: Characterization of the hidden states in the novel object assay component with respect to distances and angles (a), position of the fish in the tank (b), difference in the density of fish positions in the tank with respect to the fish position density calculated excluding the first 400 seconds of habituation (c), and hidden state usage across time (d).

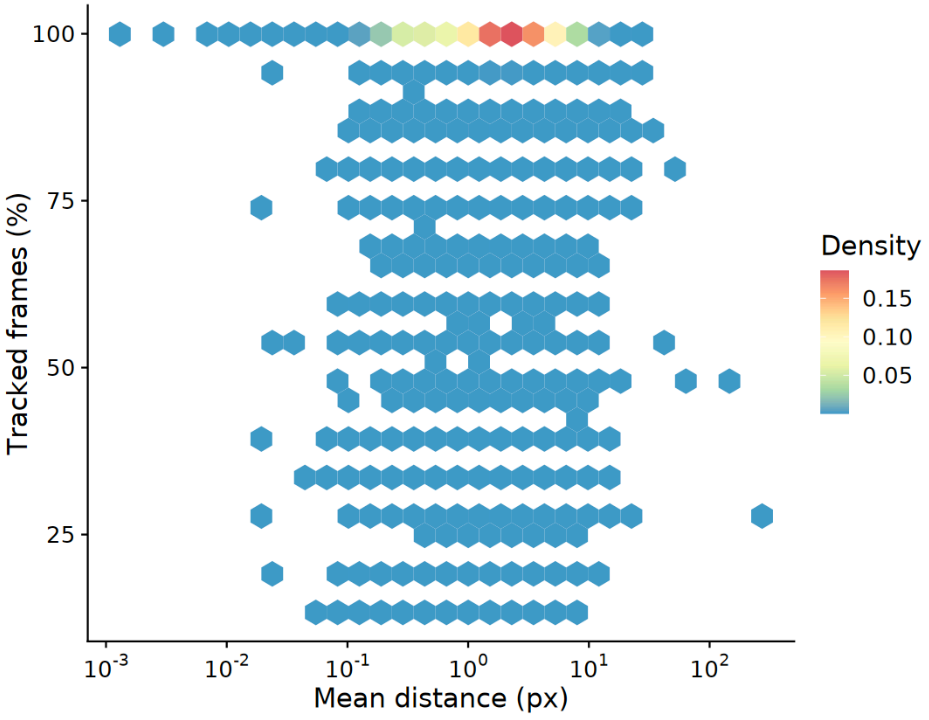

Figure S4: 2D-density plot of the mean distance travelled by the fish within 1-second segments (computed at 0.08 seconds resolution) and the percentage of video frames that failed tracking with *idtracker.ai*. The 2 variables are not correlated (Spearman ϱ = -3 * 10 -4), indicating that tracking failures do preferentially happen during fast fish movement. It was possible to compute the mean distance travelled for 99.63% of all the 1-second segments, with the remaining ones completely lacking tracked video frames.
